# Phylogenetic Characteristics and High Prevalence of a *Merbecovirus* in Hedgehogs from Greenspace of a Metropolis, China

**DOI:** 10.3390/microorganisms14010083

**Published:** 2025-12-30

**Authors:** Biao Deng, Nuo Cheng, Cheng Li, Xiao-Yang Wang, Xiao-Ling Su, Yi Sun, Jia-Fu Jiang, Luo-Yuan Xia, Wu-Chun Cao

**Affiliations:** 1 State Key Laboratory of Pathogen and Biosecurity, Beijing 100071, China; deng_biao@126.com (B.D.); cn13904296163@163.com (N.C.); licheng97@163.com (C.L.); suxll@outlook.com (X.-L.S.); sunyi7310@sina.com (Y.S.); jiangjf2008@139.com (J.-F.J.); 2 The Hospital of Yunnan Provincial Corps of the Chinese People’s Armed Police Forces, Yunnan 650100, China; 3 Institute of Pathogen Biology, Chinese Academy of Medical Sciences and Peking Union Medical College, Beijing 102629, China; 4 School of Public Health, Shandong University, Jinan 250100, China; wangxiaoyang666go@163.com

**Keywords:** *Merbecovirus*, hedgehog, urban greenspaces, viral evolution, viral recombination, cross-species transmission

## Abstract

SARS-CoV, MERS-CoV, and SARS-CoV-2 have posed tremendous threats to human health, highlighting the necessity of monitoring cross-species transmission of animal coronaviruses to humans. Hedgehogs infected with coronaviruses have been reported in several countries across Europe and Asia, raising concerns about the potential transmission of coronaviruses from hedgehogs to humans. In this study, we investigated coronavirus infections in hedgehogs inhabiting greenspaces in metropolitan Beijing and identified a *Merbecovirus* subgenus coronavirus with a prevalence rate of 30% (95% CI: 25–35%) among 317 hedgehogs. Phylogenetic analysis of 23 complete viral genome sequences revealed a monophyletic origin, showing close relatedness to *Erinaceus* hedgehog coronavirus HKU31 (*Ea*-HedCoV HKU31) with genome-wide nucleotide identities of 93.24–96.42%, and evidence of recombination with *Tylonycteris* bat coronavirus HKU4. These findings suggest that the increase in wildlife populations associated with urban greenspace development may pose a potential threat to human health that should not be overlooked.

## 1. Introduction

Coronaviruses (order *Nidovirales*, family *Coronaviridae*, subfamily *Orthocoronavirinae*) are a group of enveloped, positive-sense single-stranded RNA viruses characterized by high genetic diversity and the ability to cross species barriers. They are taxonomically classified into four genera: *Alpha*-, *Beta*-, *Gamma*-, and *Deltacoronavirus* [1]. Among these, *Alphacoronaviruses* and *Betacoronaviruses* primarily infect mammals (including humans and the bats which often serve as natural reservoirs), while *Gammacoronaviruses* and *Deltacoronaviruses* predominantly infect birds, with some capable of infecting mammals [2,3,4,5,6]. The *Betacoronavirus* genus is further subdivided into five subgenera (*Sarbecovirus*, *Merbecovirus*, *Nobecovirus*, *Hibecovirus*, and *Embecovirus*), including viruses with important clinical significance that have attracted global attention in recent years, such as the severe acute respiratory syndrome coronavirus (SARS-CoV) and SARS-CoV-2 of *Sarbecovirus*, and Middle East respiratory syndrome coronavirus (MERS-CoV) of *Merbecovirus* [7,8,9]. Bats are considered the primary natural reservoirs of CoVs, with both SARS-CoV and MERS-CoV likely originating in bats and transmitted to humans via intermediate hosts, palm civets and dromedary camels, respectively [1,10]. The host origin of SARS-CoV-2 is also suspected to involve bats and an as-yet unidentified mammalian intermediate host [11]. Increasing evidence indicates that small mammals such as hedgehogs also harbor coronaviruses with zoonotic potential [12,13].

Beijing, as one of the world’s most densely populated megacities, is home to a permanent population exceeding 20 million. Its core functional area lies within the 6th Ring Road, with the highest population density found inside the 4th Ring Road. In recent years, Beijing has witnessed continuous improvement in its urban ecological environment and steady expansion of its greenspaces. Concurrently, the diversity and abundance of urban wildlife have been on the rise. According to the Beijing Terrestrial Wildlife Checklist (2025), the city has recorded 620 species of terrestrial wildlife, belonging to 108 families across 33 orders, including 527 bird species, 63 mammal species, and 30 amphibian and reptile species (https://yllhj.beijing.gov.cn/ztxx/ysdw/ml/ (accessed on 18 December 2025)). Against this backdrop, it is increasingly important to conduct systematic surveys and research on coronaviruses carried by wildlife.

The hedgehog is a highly adaptable wild mammal that has increasingly appeared in urban greenspaces, parks, and human-inhabited areas in recent years. This urban-adaptive behavior substantially increases opportunities for contact with humans and domestic animals, thereby elevating their potential role as intermediate hosts or viral “amplifiers” [14]. Coronavirus infections in hedgehogs have been reported in multiple European countries. *Erinaceus* coronaviruses have been detected in hedgehog feces, suggesting that hedgehogs may occupy a critical position in the viral ecological network. Although infections are often subclinical, hedgehogs may still contribute to viral dissemination in urban ecosystems through environmental shedding [12,13,14,15,16,17,18,19,20,21]. In China, a few cases of coronavirus infection in hedgehogs have been reported [22,23,24]. However, no studies have investigated coronavirus infections in free-ranging urban hedgehog populations in Asia. Against the backdrop of rapid urbanization, the accelerated migration of wildlife to urban fringes and interiors has led to a sharp increase in both the diversity and abundance of urban wildlife, yet their ecological roles remain insufficiently understood. Within the framework of “One Health”, which emphasizes the interconnection between humans, animals, and the environment, viral surveillance of urban wildlife holds significant value for early public health warning.

In this study, we screened hedgehogs inhabiting urban greenspaces in metropolitan Beijing for coronavirus infection, obtained complete hedgehog coronavirus genome sequences via next-generation sequencing (NGS), and analyzed their genetic and phylogenetic characteristics. We aimed to elucidate the epidemiological and evolutionary features of hedgehog coronaviruses in the metropolitan setting, thereby providing a scientific basis for future urban ecological planning and the prevention of zoonotic diseases.

## 2. Materials and Methods

### 2.1. Sample Collection and RNA Extraction

From April to September 2024, we conducted a survey of hedgehog populations across 65 urban greenspaces in Beijing and collected swab samples from the captured hedgehogs. Based on the urban layout and transportation ring roads, the 65 surveyed greenspaces were classified into three zones: 12 sites within the 4th Ring Road, 31 sites between the 4th and 5th Ring Roads, and 22 sites outside the 5th Ring Road. Each sampling site was sampled only once, and the sampling routes did not overlap. After morphological identification, throat swabs and rectal swabs were collected from each animal using disposable viral sampling tubes (Jiangsu Kangjian Medical Apparatus Co., Ltd., Taizhou, China). After sampling, hedgehogs were released at the site of capture. Fresh samples were transported to the laboratory within a maximum of two hours and stored at −80 °C until processing. All animal handling and sampling procedures were reviewed and approved by the Laboratory Animal Use Management Committee of Laboratory Animal Center of State Key Laboratory of Pathogen and Biosecurity (approval number: IACUC-DWZX-2023-P047), and were conducted in accordance with relevant guidelines and regulations.

After thorough vortexing of the swab preservation solution, 140 µL of the supernatant was used for RNA extraction with the QIAamp Viral RNA Mini Kit (Qiagen, Hilden, Germany) according to the manufacturer’s instructions. RNA was eluted in RNase-free water and stored at −80 °C until further use.

### 2.2. Next-Generation Sequencing and Viral Genome Assembly

An initial coronavirus screening was conducted on 11 rectal swab samples by RT-PCR employing previously published primers [25]. Three samples that tested positive were further analyzed using NGS. Briefly, ribosomal RNA (rRNA) was removed using the Ribo-Zero Gold rRNA Removal Kit (Illumina, San Diego, CA, USA.). Sequencing libraries were prepared following the standard Illumina protocol and sequenced on the Illumina NovaSeq 6000 platform at Novogene company (Beijing, China) with paired-end 150 bp reads (RNA-seq). Raw Illumina reads were quality filtered using AfterQC (v2.3.3) [26] to remove low-quality and short reads. Host-derived sequences were removed by mapping the remaining high-quality reads to the European hedgehog reference genome (GCA_950295315.1_mEriEur2.1) using Bowtie2 (v2.3.5.1) [27]. Unmapped reads were *de novo* assembled using Trinity (v2.13.2) [28]. The resulting contigs were sequentially analyzed by DIAMOND BLASTx (v2.0.13) [29] against the NCBI non-redundant protein database (nr; BLAST database release, accessed 9 March 2024) and by BLASTn (v2.12.0+) [30] against the NCBI nucleotide database (nt; BLAST database release, accessed 24 January 2024). Viral-related contigs were identified based on the best homology matches, with a significance threshold of *E* < 1 × 10^−5^.

Based on one complete genome sequence obtained from the sequencing of the above three samples, primers were designed for quantitative RT-PCR assays. Samples with high viral loads were also processed using the same sequencing approach. All the obtained complete coronavirus genome sequences in this study were deposited to GenBank with accession numbers of PV953116–PV953138.

### 2.3. Phylogenetic Analysis

Multiple sequence alignments of hedgehog CoV sequences were performed using MAFFT (v7.490) [31]. Low-quality alignment regions were trimmed with TrimAl (v1.4) [32], and short contigs inconsistent with the reference genome were removed to obtain high-quality alignments for downstream analyses. The optimal amino acid or nucleotide substitution model for each dataset was determined using IQ-TREE (v2.2.2.3) [33]. Phylogenetic trees were constructed using the maximum likelihood (ML) method with 1000 bootstrap replicates. Tree visualization was conducted using the R packages ggtree (v3.8.0) [34] and ggplot2 (v3.5.1) [35].

### 2.4. Recombination Detection and Spike Protein RBD Variation Analysis

To assess potential recombination events in the genomes of hedgehog CoVs identified in this study, Bootscan analysis was performed using SimPlot (v3.5.1; SCRoftware, Johns Hopkins University School of Medicine, Baltimore, MD, USA) with a 1000 bp sliding window and 200 bp step size, based on the Kimura two-parameter model and the Neighbor-Joining method. Recombination signals were evaluated with 100 bootstrap replicates. For spike protein variation analysis, amino acid sequences of the receptor-binding domain (RBD) were aligned with MAFFT (v7.490) (as above). Key receptor-binding residues were manually inspected based on known MERS-CoV RBD interaction sites, and important amino acid substitutions or insertions were annotated.

### 2.5. Quantitative Real-Time RT-PCR Detection

Specific primers (5′-ACCCATGCTCCATTCCCAAG-3′ and 5′-TGGCWACTCCACTTCCACAG-3′) and a fluorescent probe (AGGGGTTCTAGTGTAAATGCCCGACT) were designed based on the initial sequencing result of the CoV genome sequence from a hedgehog in this study. One-step quantitative real-time RT-PCR was conducted to detect hedgehog CoVs. The reaction conditions were as follows: 42 °C for 5 min, 95 °C for 10 s, followed by 40 cycles of 95 °C for 5 s and 60 °C for 34 s. Nuclease-free water was included as a negative control in each run to avoid false positives and PCR contamination. To construct the standard curve for hedgehog CoV quantitative RT-PCR, 10-fold serial dilutions of quantified RNA standards were prepared. The standard curve exhibited a correlation coefficient (R^2^) of 0.9998 and an amplification efficiency of 97%. The limit of detection (LOD) was determined to be approximately 56 copies/µL. The copy number of hedgehog CoV in each sample was calculated by applying the corresponding Ct values to the standard curve.

### 2.6. Statistical Analyses

Positivity rates were expressed as percentages with 95% confidence intervals (95% CI) calculated using the Wilson score method, which provides more accurate estimates, particularly for small sample sizes or extreme proportions. Differences in positivity rates between groups were evaluated using Chi-square tests (*χ*^2^) [36], and Fisher’s exact test was applied when expected cell counts were low. The Cochran–Armitage trend test was employed to assess the significance of trends. All statistical analyses were conducted in R software, with two-sided tests and a significance threshold of *p* < 0.05.

## 3. Results

### 3.1. Field Survey

From April to September 2024, a total of 317 hedgehogs were captured from 65 urban greenspaces in Beijing, and 312 rectal swabs and 317 throat swabs collected. Morphological observation identified all individuals as Amur hedgehogs (*Erinaceus amurensis*). The number of hedgehogs captured per site ranged from 0 to 15, with a median of 4. Sex was recorded for 310 individuals, comprising 123 females and 184 males. Hedgehog presence was observed at all but one sampling site.

### 3.2. Positive Rate of Coronavirus in Hedgehogs

**Figure 1:**
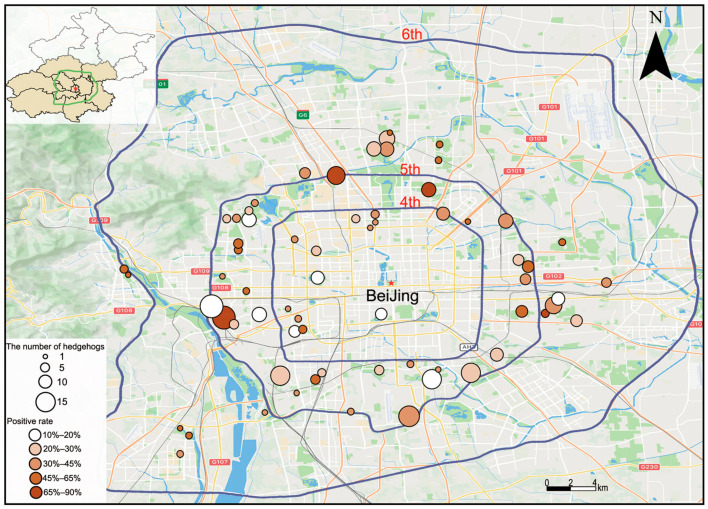
Survey sites of hedgehogs in greenspaces in Beijing and the coronavirus positive rates. Map of Beijing illustrating the locations of the 65 sampling sites. The circle size indicates the number of hedgehog samples collected from each greenspace, and the circle color indicates the coronavirus infection rate in hedgehogs. The red star represents the central location of Beijing.

From the initial sequencing of three rectal swab samples, one complete coronavirus genome was obtained. Sequence alignment revealed that the sequence was identified as *Erinaceus* hedgehog coronavirus HKU31 (*Ea*-HedCoV HKU31). All hedgehog specimens were screened by quantitative RT-PCR using HKU31-specific primers mentioned in the Methods section. Across the 65 surveyed urban greenspaces, HKU31 RNA was detected in rectal swab samples from 95 hedgehogs at 36 sites with a mean viral RNA of 2.28 × 10^6^ copies/µL (95% CI: 6.22 × 10^5^–3.96 × 10^6^). In addition, one throat swab tested positive with the viral RNA of 1.29 × 10^4^ copies/µL, which corresponded to an individual who was also positive in the rectal swab. Based on the 312 rectal swabs tested, the overall prevalence was 30% (95% CI: 25–35%) (Figure 1).

No significant difference in positive rate was observed between females (24.39%) and males (33.15%) (*χ*^2^ test, *p* = 0.097). However, prevalence differed significantly among geographic zones, and a Cochran–Armitage test for trend indicated a significant association between positive rate and geographic location (*χ*^2^ for trend, *p* = 0.007). The positive rate of coronavirus exhibited a clear geographic pattern, with significantly lower positive rate within the 4th Ring Road compared to the 4th–5th Ring Road zone (*p* < 0.001) and outside the 5th Ring Road (*p* < 0.001). This suggests that hedgehogs closer to the city center have a lower infection rate. The positive rate also varied by sampling period: the prevalence in April–May was significantly lower than in June–July (*p* < 0.001) and August–September (*p* < 0.001), whereas no significant difference was found between June–July and August–September (both *p* > 0.05) (Table 1).

### 3.3. Phylogenetic Analysis of Hedgehog Coronavirus Based on Whole Genomes

**Figure 2:**
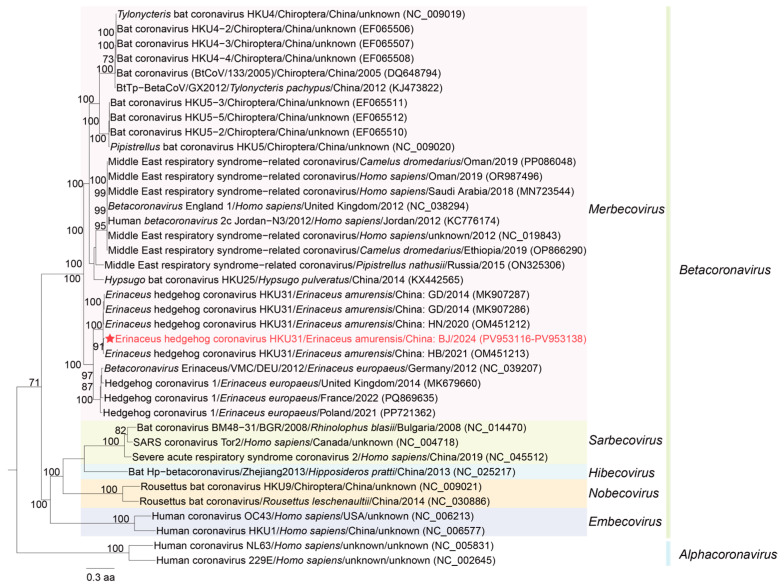
Phylogenetic analysis of the ORF1ab protein sequences of *Betacoronavirus* and *Alphacoronavirus* was performed using the maximum likelihood (ML) method to infer the evolutionary relationships of BJ-HKU31. Branch support values were assessed using 1000 bootstrap replicates, and only bootstrap values greater than 70 are shown on the tree. The phylogenetic tree was annotated with information on the organism, host, collection region, and collection date. Sequences generated in this study are indicated by red stars. Light pink represents *Merbecovirus*, light yellow represents *Sarbecovirus*, light blue represents *Hibecovirus*, light orange represents *Nobecovirus*, and light purple represents *Embecovirus*.

Whole genome sequencing was performed on samples with high viral load detected via quantitative RT-PCR, an additional 21 genomes were obtained from rectal swabs, and 1 genome was obtained from throat swabs. Together with the whole genome obtained from the initial sequencing round, a total of 23 complete coronavirus genomes were assembled in this study (GenBank accession number PV953116–PV95338), showing whole-genome nucleotide identities ranging from 93.24 to 99.99%. Phylogenetic analysis revealed (Figure 2) that the coronaviruses detected in hedgehogs from Beijing were most closely related to the HB-HKU31 strain identified in hedgehogs from the neighboring province of Hebei (GenBank accession number: OM451213). The whole-genome nucleotide identities between these viruses ranged from 95.56 to 96.42%, and the ORF1ab amino acid identities ranged from 97.44 to 98.50%, with these strains clustering tightly within the same clade of the phylogenetic tree. In addition, HKU31 strains derived from hedgehogs in Beijing and Hebei formed a clade together with the *Ea*-HedCoV HKU31 strains from Guangdong Province (GenBank accession numbers: MK907286 and MK901287) and the HN-HKU31 strain from Henan Province (GenBank accession number: OM451212).

### 3.4. Analysis of the BJ-HKU31 Spike Protein

**Figure 3:**
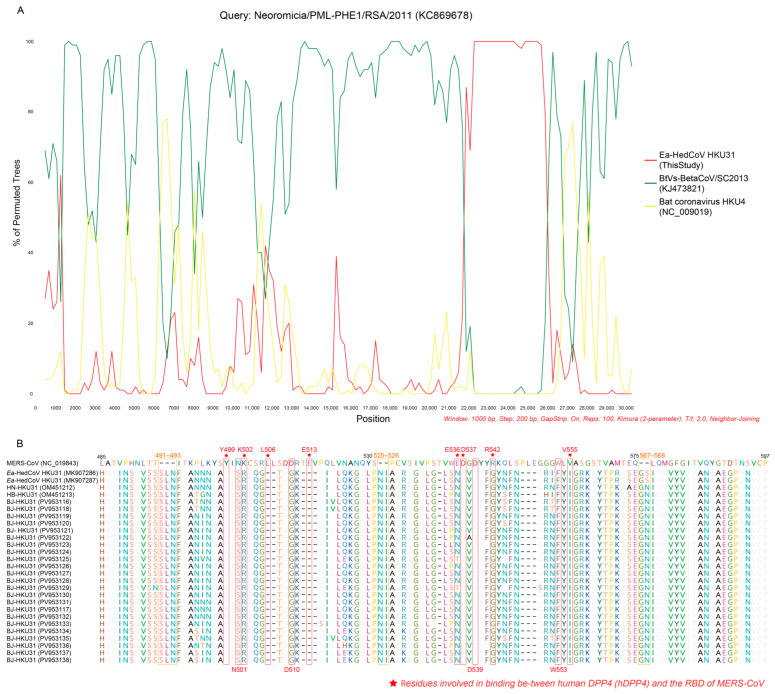
Analysis of the BJ-HKU31 spike (S) protein. (**A**) Detection of a possible recombination event was conducted using bootscan analysis. A whole genome sequence similarity plot was performed using Simplot (v3.5.1), with a window size of 1000 bp and a step size of 200 bp. A bat coronavirus (GenBank accession no. KC869678) served as the query sequence and was compared against other coronaviruses, including *Ea*-HedCoV HKU31 (red, this study), BtVs-BetaCoV/SC2013 (green), and bat coronavirus HKU4 (yellow). (**B**) Amino acid sequence alignments of receptor-binding domain (RBD) sequences of hedgehog coronaviruses. The sequence of MERS-CoV (GenBank accession no. NC_019843) was used as the reference sequence. Different colors are used to highlight distinct residues, red asterisks mark conserved residues (functionally critical in MERS-CoV).

Previously, it was reported that the *Ea*-HedCoV HKU31 strain identified in Guangdong exhibited recombination with bat coronaviruses in the spike (S) protein, particularly in the S1 region [21]. Recombination analysis of the BJ-HKU31 genomes in this study similarly revealed and confirmed a recombination event in the S protein region (approximately 22,000–26,000 bp) with a bat coronavirus (GenBank accession no. KC869678) (Figure 3A).

The RBD of the coronavirus S protein is a key determinant of viral entry into host cells and a primary target for neutralizing antibodies [37]. MERS-CoV utilizes the dipeptidyl peptidase-4 (DPP4) receptor for host cell entry, and amino acid variations in the RBD can significantly influence viral transmissibility, pathogenicity, and immune evasion. Previous studies have identified 12 critical residues involved in binding between human DPP4 (hDPP4) and the RBD of MERS-CoV, namely Y499, N501, K502, L506, D510, E513, E536, D537, D539, R542, W553, and V555 [38,39]. Using the MERS-CoV sequence (GenBank accession no. NC_019843) as a reference, analysis of these residues in the BJ-HKU31 RBD revealed that only Y499, D537, and D539 were conserved (Figure 3B).

## 4. Discussion

This study, combining surveys of urban landscapes, found that the prevalence of CoV in hedgehogs in Beijing metropolitan reached 30%. Hedgehogs serve as a potential viral reservoir for human pathogens [40]. Previous studies have shown that hedgehogs are natural hosts of CoVs in both Europe and Asia. In Europe, the main host is the European hedgehog, in which EriCoV is widely distributed, although prevalence exhibits significant regional variation. In 2013, a novel *Betacoronavirus* closely related to MERS-CoV and lineage C bat coronaviruses was detected in European hedgehogs in northern Germany [12]. Subsequently, hedgehog coronavirus infections were reported in the United Kingdom, France, Italy, Poland, Portugal, Hungary, and Russia [13,14,15,16,17,18,19,20,21], with Portugal additionally reporting the first detection of an alphacoronavirus in hedgehogs, closely related to *Miniopterus* bat coronavirus HKU8.

Phylogenetic analysis revealed that BJ-HKU31, identified in this study, clusters together with HKU31 reported from other regions of China and with Hedgehog coronavirus 1 detected in Europe. These viruses constitute a hedgehog-associated coronavirus lineage, which is distinct from the *Merbecovirus* lineages found in humans, bats, rodents, and other mammalian hosts. Since hedgehogs and bats both belong to the order Eulipotyphla, the detection of recombination events between BJ-HKU31 and bat-borne coronaviruses highlights the importance of monitoring coronavirus recombination and the potential risk of cross-species transmission.

Previous studies analyzing the spike protein of HKU31 through sequence analysis and structural modeling indicated that this hedgehog coronavirus is unlikely to bind human DPP4 [22]. Similar conclusions were drawn from sequence and structural analyses of EriCoV spike proteins from Italy [16]. However, studies simulating interactions between the hedgehog coronavirus RBD and ACE2 receptors from hedgehogs and other potential animal hosts predicted the strongest binding affinity between the hedgehog coronavirus RBD and human ACE2, with binding to hedgehog ACE2 stronger than to hedgehog DPP4 [21]. Using pseudovirus systems, HKU31 infectivity was assessed in RBP-expressing cells, showing that HKU31 is unlikely to enter human cells. Nevertheless, after trypsin pre-treatment of HKU31 pseudoviruses, Western blot analysis confirmed spike cleavage, enabling HKU31 to infect 18 of 51 NCI-60 cell lines [41]. HKU31 was also detected in deceased hedgehogs collected in Hebei Province, China, with scattered pulmonary calcifications observed, suggesting HKU31 pathogenicity in hedgehogs [23]. In the present study, we identified mutations at multiple sites in the RBD region that differ from the four previously reported HKU31 sequences. Although the functional implications of these mutations are currently unknown, they provide a reference for investigating hedgehog coronavirus transmission characteristics. Y499 is part of the hydrogen bond interaction between the MERS-CoV RBD and hDPP4. D537 and D539 contribute to the formation of a negatively charged surface, with D539 capable of forming a salt bridge with a basic residue in DPP4. These residues play critical roles in receptor binding and viral entry. In contrast, amino acid substitutions were observed at N501, K502, D510, E536, R542, W553, and V555, while deletions were detected at L506 and E513. Amino acid insertions occurred at positions 491–493, 525–526, and 567–568. Although the biological significance of these variations remains unclear, they suggest a potential for cross-species transmission of hedgehog CoVs.

This study obtained 23 complete HKU31 genomes, increasing the total number of complete sequences from four to 27. The sequences generated here exhibit high internal homology and are closely related to previously reported hedgehog CoVs. Notably, European hedgehogs are primarily infected with EriCoV, whereas northeastern Chinese hedgehogs are mainly infected with HKU31. Moreover, evolutionary differentiation was observed between HKU31 CoVs from northern and southern China, indicating host species- and geographic-specific variation in hedgehog coronavirus infection.

Urban greenspaces serve as concentrated areas for outdoor recreation, where human and domestic animal (e.g., cats and dogs) contact with hedgehogs is frequent. In China, it is common for people to walk in parks after dinner. Notably, cats are widespread in greenspaces, and we observed hedgehogs and cats feeding together on food provided by humans. Comparative analyses of the hedgehog CoV RBD and DPP4 receptors from humans, dogs, cats, foxes, badgers, and hedgehogs predicted the strongest binding to fox and cat DPP4, suggesting a higher risk of cross-species transmission to these animals [21]. The potential spillover of HKU31 to other urban animals warrants further investigation.

In densely populated metropolises like Beijing, pathogens can rapidly spread among human populations once introduced. Infected individuals or contaminated objects (e.g., goods) can facilitate rapid transmission to other cities or globally via air and land transportation. Therefore, continuous monitoring of urban wildlife for pathogens is essential. It is particularly important to track the evolution of hedgehog CoVs to anticipate potential recombination events with other CoVs (e.g., bat- or other animal-derived), which could generate novel viruses with interspecies transmission potential.

Isolation of hedgehog CoVs and *in vitro* experiments to evaluate their transmissibility and pathogenicity are critical. To date, no successful isolation of hedgehog coronaviruses has been reported. Additionally, we did not assess the age or health status of hedgehogs during sample collection, limiting our ability to evaluate age-related susceptibility or the impact of coronavirus infection on hedgehog health.

## 5. Conclusions

In summary, this study provides the first description of the widespread distribution and high prevalence of coronavirus in hedgehogs within urban greenspaces of a megacity. It reveals a gradient evolution of coronavirus infection during hedgehog urban migration and highlights host species- and geographic-specific differences in infection. Furthermore, mutations in key residues of BJ-HKU31 RBD were characterized. The high prevalence of BJ-HKU31 in urban greenspaces hedgehogs underscores the potential risk of pathogen transmission from wildlife to human-populated areas as urban ecosystems improve, emphasizing the importance of monitoring and studying urban wildlife.

## Figures and Tables

**Table 1:** Statistical analysis of BJ-HKU31 infection rates in hedgehogs. Incorporating sex, region, and sampling month as categorical factors to examine differences in infection rates.

Category	Sub-Category	No.	No. of Positive	Positive Rate (%, 95% CI)	*p*
Sex	Male	184	61	33.15 (26.76–40.24)	0.097
	Female	123	30	24.39 (17.65–32.68)	
Region	Within the 4th Ring Road	36	2	5.56 (1.54–18.14)	<0.001
	4th–5th Ring Road	171	56	32.75 (26.16–40.09)	
	outside the 5th Ring Road	108	37	34.26 (25.99–43.61)	
Month	April and May	90	8	8.89 (4.57–16.57)	<0.001
	June and July	145	59	40.69 (33.03–48.83)	
	August and September	82	28	34.15 (24.8–44.91)	

## Data Availability

The original contributions presented in this study are included in the article. Further inquiries can be directed to the corresponding authors.

## Abbreviations

The following abbreviations are used in this manuscript:

SARS-CoV	Severe Acute Respiratory Syndrome Coronavirus
MERS-CoV	Middle East Respiratory Syndrome Coronavirus
SARS-CoV-2	Severe Acute Respiratory Syndrome Coronavirus 2
COVID-19	Coronavirus Disease 2019
CoVs	Coronaviruses
HKU4	Bat Coronavirus HKU4
*Ea*-HedCoV HKU31	*Erinaceus* hedgehog coronavirus HKU31
HKU8	Bat Coronavirus HKU8
EriCoV	*Erinaceus* Coronavirus
S	Spike
RBD	Receptor-Binding Domain
ACE2	Angiotensin-Converting Enzyme 2
DPP4	Dipeptidyl Peptidase 4
BJ-HKU31	Beijing *Ea*-HedCoV HKU31
HB-HKU31	Hubei *Ea*-HedCoV HKU31
HN-HKU31	Henan *Ea*-HedCoV HKU31
